# Acute bowel ischemia in the absence of radiological findings: A case for endoscopy

**DOI:** 10.1097/MD.0000000000050638

**Published:** 2026-09-11

**Authors:** Patricia Mester, Arne Kandulski, Florian Weber, Stephan Schmid, Martina Müller, Vlad Pavel

**Affiliations:** a Department of Internal Medicine I, Gastroenterology, Hepatology, Endocrinology, Rheumatology, and Infectious diseases, University Hospital Regensburg, Regensburg, Germany; b Institute of Pathology, University of Regensburg, Regensburg, Germany.

**Keywords:** Bowel ischemia, endoscopy, sepsis

## Abstract

**Rationale:**

In cases of suspected acute mesenteric ischemia, current guidelines recommend computed tomography angiography as the initial diagnostic modality, with direct laparoscopy advised in uncertain cases. However, the occurrence of nontherapeutic laparoscopies remains a clinical concern. Notably, endoscopy is not yet incorporated into standard diagnostic algorithms.

**Patient concerns:**

Here, we report the case of a 73-year-old man admitted to the intensive care unit in septic shock. Laboratory tests showed markedly elevated inflammatory markers, creatine kinase, and lactate levels. His medical history included a large chronic left-sided inguinoscrotal hernia, which was reducible and painless at admission. Both clinical symptoms and radiological findings were inconclusive for bowel ischemia.

**Diagnoses:**

Since both clinical symptoms and radiological findings were unspecific, endoscopy was performed, and identified transmural bowel ischemia, highlighting its potential role as a valuable, minimally invasive tool in the diagnostic pathway for intestinal ischemia.

**Interventions:**

In uncertain cases, current guidelines recommend diagnostic laparotomy to diagnose bowel ischemia. However, unnecessary or nontherapeutic laparotomies are a serious concern, which can lead to morbidity and even death. Gastrointestinal endoscopy is a common procedure available worldwide. Despite its safety and availability, it is not mentioned in the current guidelines for the diagnosis of bowel ischemia.

**Outcomes and lessons:**

Although the patient died due to his several comorbidities, this case demonstrates that endoscopy can detect intestinal ischemia even when radiological findings are inconclusive. This case clearly suggests that endoscopy should be incorporated into the diagnostic algorithm in patients with suspected mesenteric ischemia.

## 1. Introduction

Acute mesenteric ischemia (AMI) is a life-threatening condition characterized by a high mortality rate.^[1]^ Cokkinis expressed profound pessimism already almost a century ago regarding this condition, when he wrote “’the diagnosis is impossible, the prognosis hopeless and the treatment almost useless”’.^[2]^ Although significant diagnostic and therapeutic advances have been achieved since then in the medical field, identification and timely intervention remain challenging nowadays.^[3]^

AMI accounts for 0.09% to 0.2% of all acute admissions to emergency departments but represents a common cause of urgent intestinal resection.^[4]^ Late diagnosis or untreated lesions lead to mesenteric infarction, bowel necrosis and can progress to sepsis and death.^[1]^ Timely intervention can stop and reverse this process, leading to clinical improvement and survival. However, diagnosis of AMI remains difficult and can be easily overlooked.^[1,3]^ Several pathological etiologies are responsible for the development of bowel ischemia and necrosis, dividing this condition into different subtypes.^[4,5]^ 50% of these cases are caused by emboli affecting the superior mesenteric artery.^[4]^ Intracardiac emboli or an atherosclerotic aorta could be the source of them. Arterial thrombosis is another mechanism that can induce mesenteric ischemia. This pathology usually occurs at the origin of visceral arteries. Another entity is represented by nonocclusive mesenteric ischemia (NOMI), which occurs in about 20% of cases. Cardiac failure, sepsis, vasoactive agents and hypovolemia are risk factors for NOMI. In <10% of cases, venous thrombosis is responsible for bowel infarction. Stagnant blood flow, hypercoagulability, and endothelial damage may lead to thrombosis. These changes can occur in diseases like Factor V Leiden, prothrombin mutation, protein S deficiency, protein C deficiency, antithrombin deficiency, or antiphospholipid syndrome.^[4]^

The diagnosis of AMI is frequently delayed owing to its nonspecific clinical and laboratory features, leading to the description “The Chameleon of the Acute Abdomen.”^[3]^ Although abdominal pain is a common presenting symptom, signs of peritonitis are often subtle or absent. Associated symptoms such as nausea, vomiting, diarrhea and, occasionally, hematochezia may raise clinical suspicion.^[4]^ In critically ill or sedated patients, reliable symptom assessment is further complicated. While elevated serum lactate levels may support the diagnosis, normal values do not exclude AMI,^[5]^ and no specific biomarker currently exists to reliably confirm or rule out transmural ischemia.^[4]^

Current guidelines recommend urgent computed tomography angiography (CTA) in all suspected cases.^[4]^ Radiological features suggestive of transmural ischemia include pneumoperitoneum, lack of bowel wall enhancement, and pneumatosis intestinalis.^[5]^ However, these findings lack specificity, as demonstrated by a multicentric study in which 60% of patients with pneumatosis intestinalis did not have underlying bowel ischemia.^[6]^

Although routine use of diagnostic laparoscopy is not recommended, current guidelines acknowledge its role in uncertain cases, even in the intensive care setting.^[1]^ Nevertheless, unnecessary or nontherapeutic laparotomies continue to be reported in patients with suspected AMI.^[7,8]^

American guidelines advocate for early colonoscopy in cases of suspected colonic ischemia, particularly when CT angiography reveals bowel wall thickening, although this recommendation is supported by low-quality evidence.^[9]^

Despite being widely available, minimally invasive, and well suited to examine nearly the entire gastrointestinal tract, endoscopy is not currently incorporated into guideline-based diagnostic algorithms for AMI.^[4]^

## 2. Case report

**Figure 1.**
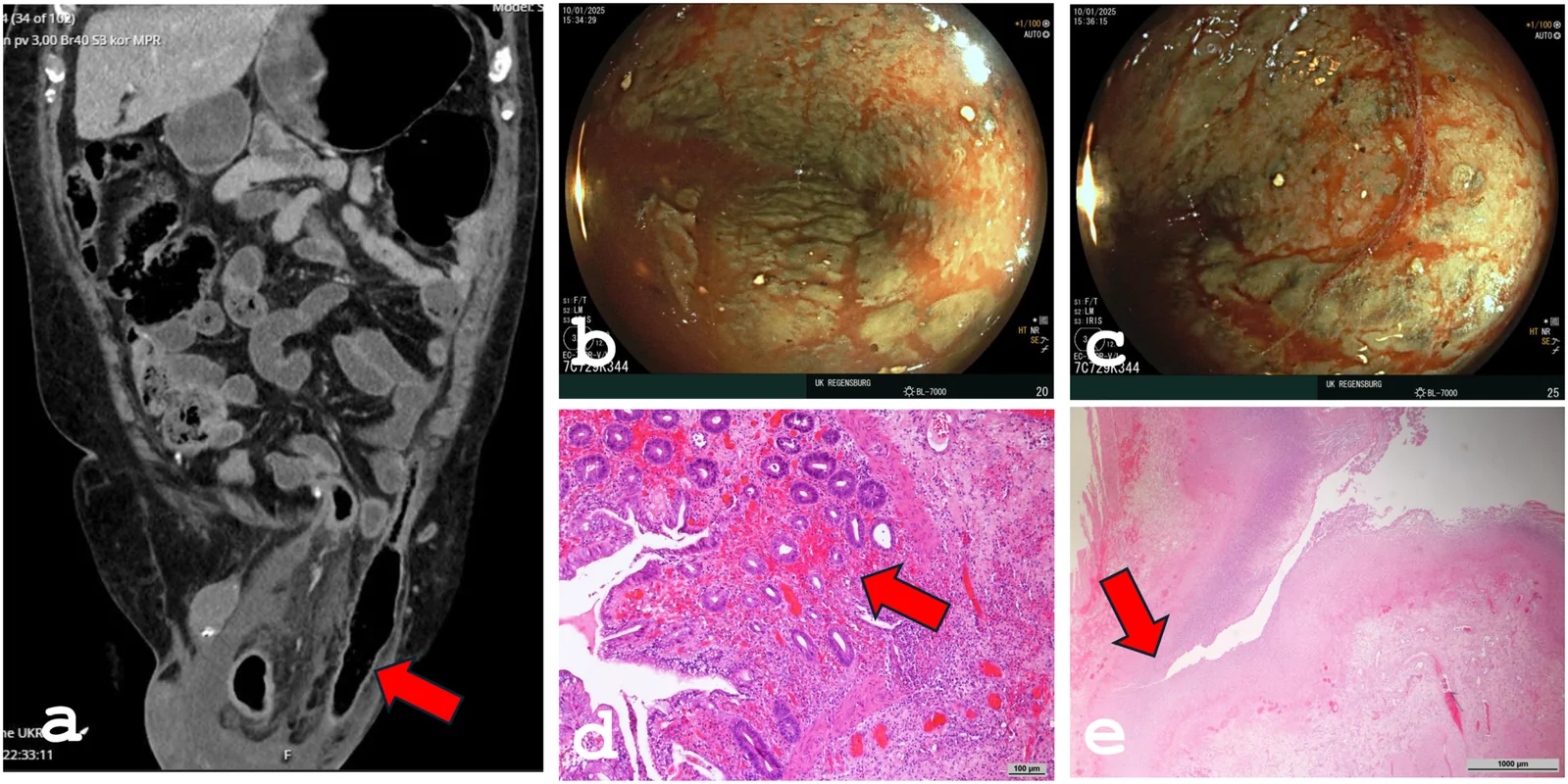
Contrast-enhanced CT scan showing a large left-sided inguinoscrotal hernia with herniation of bowel loops, mesenteric fat stranding, and ascites, but no pneumatosis intestinalis (arrow) (A). Endoscopic views showing extensive, circumferential mucosal necrosis of the sigmoid colon, indicative of transmural ischemia (B, C). Histopathological analysis of the surgical specimen confirmed acute ischemic colitis, characterized by crypt loss (arrow), hemorrhage, epithelial destruction, and neutrophilic infiltration of the submucosa (D) with evidence of transmural extension (arrow) of the ischemic injury (E) (hematoxylin and eosin stain, original magnification × 100).

We describe the case of a 73-year-old man admitted to the intensive care unit in septic shock. On examination, he was hypotensive (70/40 mm Hg) with reduced consciousness but no abdominal complaints. His medical history included advanced chronic obstructive pulmonary disease (according to the Global Initiative for Chronic Obstructive Lung Disease stage 4), severe pulmonary hypertension, atherosclerosis, and a large chronic left-sided inguinoscrotal hernia. Laboratory tests showed markedly elevated inflammatory markers (leukocytes 19.6/nl, CRP 125 mg/L, PCT > 100 ng/ml), creatine kinase (626 U/L), and lactate (40 mg/dL). There were no clinical signs of hernia incarceration or strangulation. CTA was performed due to persistent septic shock and revealed herniated intestinal loops within the hernial sac, mesenteric fat stranding, and ascites, in the context of early-stage liver cirrhosis. Pneumatosis intestinalis was absent (Fig. 1A). Broad-spectrum antibiotics were initiated, but the patient remained septic with persistently elevated inflammatory markers. To directly evaluate this region, sigmoidoscopy was performed 2 hours after admission to the ICU and revealed extensive circumferential mucosal necrosis of the sigmoid colon, suggestive of transmural ischemia (Fig 1A–C). Emergency surgery confirmed these findings, and a segmental colectomy with descendostomy was performed. Histopathological analysis demonstrated classical features of acute ischemic colitis – including crypt loss, hemorrhage, epithelial necrosis, and submucosal neutrophilic infiltration (Fig. 1D) – with confirmation of transmural involvement (Fig. 1E). Postoperatively, inflammatory markers declined, and vasopressor support was reduced. Nevertheless, due to the patient’s severe comorbidities, clinical recovery was not achieved, and he died 2 weeks later, during the intensive care stay.

In the present case, transmural ischemia of the sigmoid colon was confirmed by endoscopy, while computed tomography angiography showed no specific signs of bowel injury. Despite broad-spectrum antibiotic therapy and advanced supportive care, the patient did not recover due to multiple comorbidities. Endoscopy enabled direct visualization of the ischemic lesions and guided the surgical approach. No signs of bowel wall compromise were evident on imaging.

Although the patient in this case suffered from a large chronic left-sided inguinoscrotal hernia, which at the time of admission was reducible and painless, the hernia probably exerted a certain degree of strangulation which caused the secondary ischemic lesions.

## 3. Discussions

Due to nonspecific clinical and laboratory features, the diagnosis of AMI is often delayed. In suspected cases, CTA is recommended by the current guidelines.^[4]^ However, radiological findings are unspecific for the diagnosis of bowel ischemia and are encountered in several other pathologies.^[6,10–12]^ For example, pneumatosis intestinalis, which has a specificity for bowel ischemia that approaches 100%,^[13]^ is encountered in several other benign pathologies like bowel infection, trauma, chronic obstructive pulmonary disease, immunosuppressive therapy, or other medication.^[10]^ Bowel wall hypoenhancement and thickening are also encountered in other pathologies like radiation enteritis or amyloidosis.^[14]^ Bowel dilatation may occur in obstructive or nonobstructive etiologies, as described by Mari and Emmanuel.^[15]^ Finally, mesenteric edema or stranding may be caused, besides bowel ischemia, also by acute diverticulitis, colon cancer with perforation, or infectious enteritis and colitis.^[16]^

In uncertain cases, current guidelines postulate that diagnostic laparotomy should be performed.^[1]^ However, unnecessary or nontherapeutic laparotomies are a clinical concern.^[7,8]^ This fact is supported by several studies. For example, Groteleuschen et al showed that in a cohort of 275 patients with suspected AMI, in 75 cases the diagnosis was not confirmed during laparotomy. Since explorative laparotomy is the only reliable diagnostic method to detect mesenteric infarction so far, the authors conclude that new diagnostic parameters are needed.^[3]^ This concern was highlighted by further investigators. In some cohorts, more than 50% of cases are ultimately not confirmed with the disease.^[17,18]^ Bourcier et al suggest performing digestive explorations by endoscopy and/or laparotomy in case of high clinical suspicion.^[18]^ A multicenter study conducted by Andrei et al concludes that only explorative laparotomy should be performed in suspected cases.^[17]^ On the other hand, Emile et al revealed a mortality of 12.5% and a morbidity of 50% for patients receiving nontherapeutic laparotomy.^[19]^

Although is widely available, less invasive, and suited to investigating almost the entire gastrointestinal tract, endoscopy is currently not included in the guideline-based diagnostic algorithms for AMI.^[4]^ Solely, American guidelines recommend colonoscopy in cases of suspected colonic ischemia, particularly when CT angiography reveals bowel wall thickening, though this recommendation is supported by low-quality evidence.^[9]^

Mucosal necrosis does not necessarily indicate transmural ischemia; however, gray-green or even black mucosal appearance may suggest transmural infarction.^[20,21]^ Moreover, this endoscopic finding correlated with clinical sepsis features could indicate transmural bowel ischemia.^[22,23]^

Historically, some physicians were reticent regarding endoscopy in cases with suspected bowel ischemia due to perforation risk.^[24]^ However, recent data highlights the safety of this procedure in this setting, even in critically ill patients.^[25,26]^ Since bowel ischemia is an emergency, colonoscopy is mostly performed without adequate prior bowel preparation in these situations. To minimize the risk of perforation, the procedure should be performed by experienced endoscopists.^[25]^

**Figure 2.**
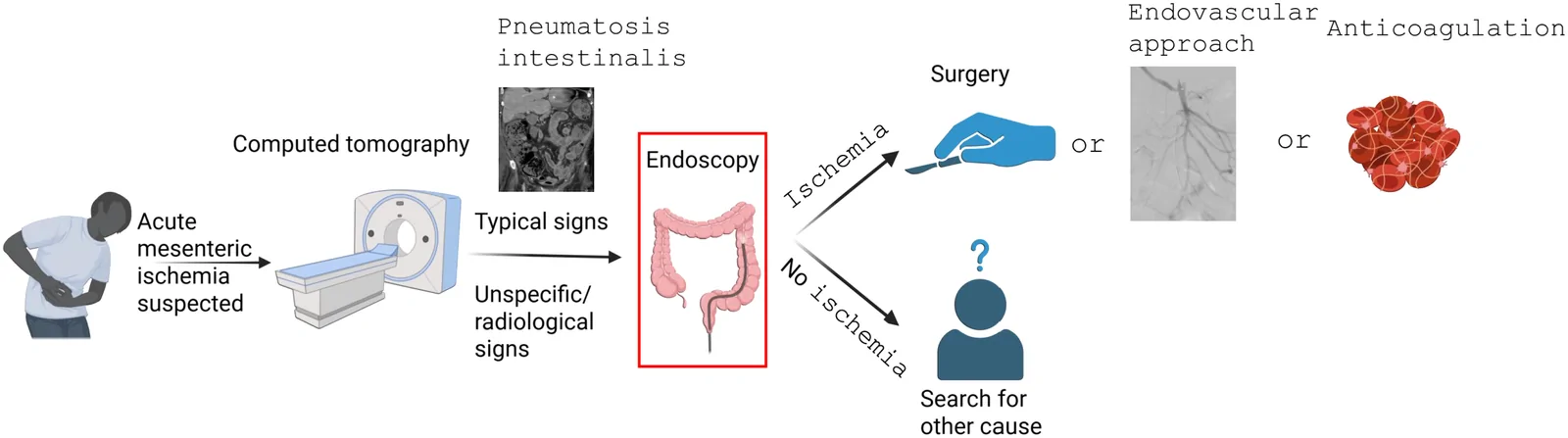
Diagnostic algorithm for patients with suspected bowel ischemia (created with BioRender).

Taking altogether, previous experience with this pathology suggests that new investigations should be introduced in clinical practice for better management of patients with suspected bowel ischemia. Given the continuous improvements in endoscopic safety measures,^[27]^ even in patients with ischemic colonic lesions,^[26]^ gastrointestinal endoscopy could represent an accurate tool in diagnosing or ruling out bowel ischemia. Therefore, endoscopy should be at least mentioned as an additional investigation in unclear cases, when clinical symptoms and radiological findings are inconclusive. Figure 2 illustrates a new possible algorithm that includes endoscopy for patients with suspected mesenteric ischemia. This algorithm recommends performing endoscopy before proceeding to surgical exploration in uncertain cases. This approach could avoid nontherapeutic laparotomies.

## 4. Conclusion

This case demonstrates that endoscopy can detect intestinal ischemia even when radiological findings are inconclusive. It offers a minimally invasive diagnostic option and may prevent unnecessary surgical interventions. Given its diagnostic precision, endoscopy should be considered for inclusion in clinical algorithms for the assessment of suspected mesenteric ischemia.

## Author contributions

**Conceptualization:** Patricia Mester, Arne Kandulski, Florian Weber, Martina Müller, Vlad Pavel.

**Data curation:** Patricia Mester, Arne Kandulski, Florian Weber, Stephan Schmid, Martina Müller, Vlad Pavel.

**Writing – original draft:** Patricia Mester, Vlad Pavel.

**Supervision:** Arne Kandulski, Martina Müller.

**Investigation:** Stephan Schmid.

**Methodology:** Stephan Schmid.
